# Supplementary material for: Simplicial models of social contagion
Source: Nat Commun. 2019 Jun 6;10:2485. doi: 10.1038/s41467-019-10431-6 (PMC6554271; doi:10.1038/s41467-019-10431-6)
Supplement: Supplementary file 1 — Supplementary Information [file 41467_2019_10431_MOESM1_ESM.pdf]

# Supplementary Information

# "Simplicial models of social contagion"

Iacopini et al.

## Supplementary Note 1. GENERALIZED DEGREE DISTRIBUTIONS OF EMPIRICAL AND SYNTHETIC SIMPLICIAL COMPLEXES

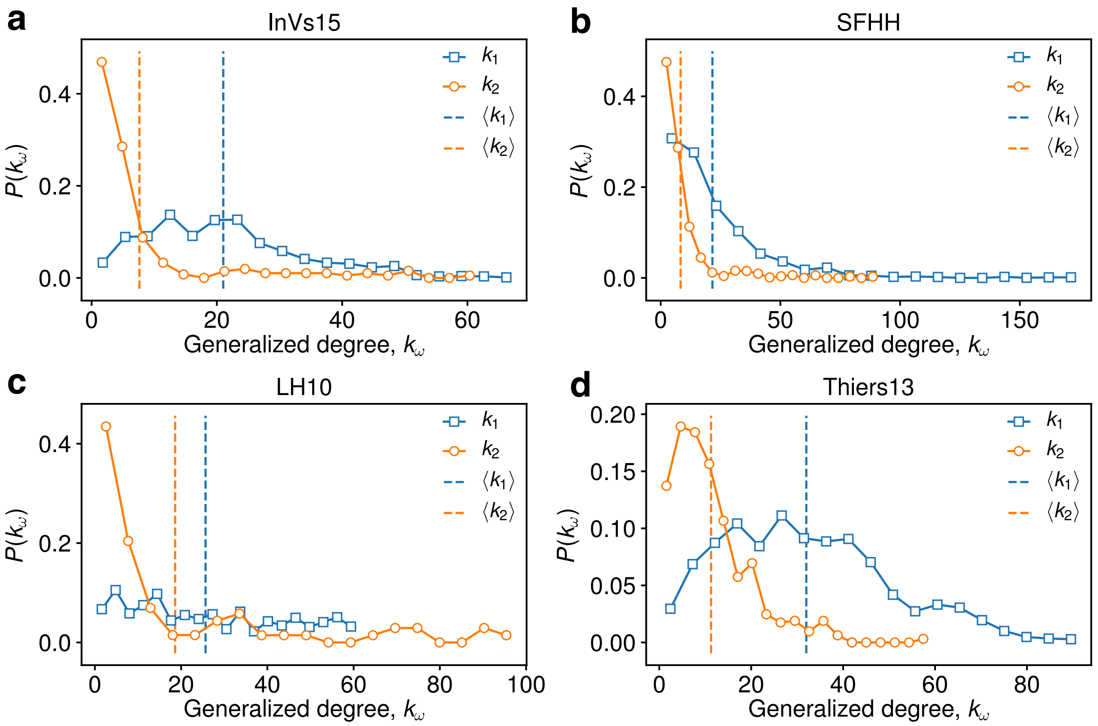


**Supplementary Figure 1.** Generalised degree distributions of random simplicial complexes created from real world data sets (see the data processing method described in the "Methods" section of the main text). The four panels correspond to different social contexts, namely (**a**) a workplace (InVS15), (**b**) a conference (SFHH), (**c**) a hospital (LH10) and (**d**) a high school (Thiers13). The generalised degrees $k_1$ and $k_2 = k_\Delta$ denote respectively the number of 1-simplices (blue) and 2-simplices (orange) incident in a node. The vertical dashed lines indicate the corresponding average values.

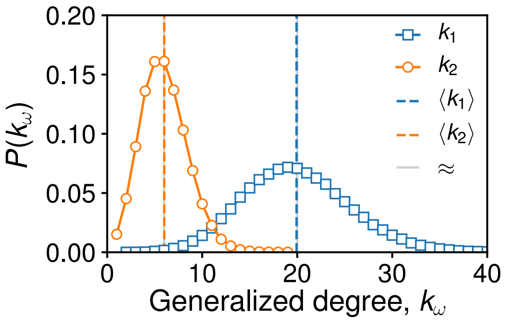


**Supplementary Figure 2.** Generalised degree distributions of random simplicial complexes (RSC) generated by the model described in the main text. The generalised degrees $k_1$ and $k_2 = k_\Delta$ denote respectively the number of 1-simplices (blue) and 2-simplices (orange) incident in a node. The vertical lines compare the average values of $\langle k_1 \rangle$ and $\langle k_2 \rangle$ obtained from multiple realizations of the model (coloured dashed lines) with the approximated values (continuous grey lines) calculated as described in the main text.

## Supplementary Note 2. HYSTERESIS AND SYSTEM SIZE

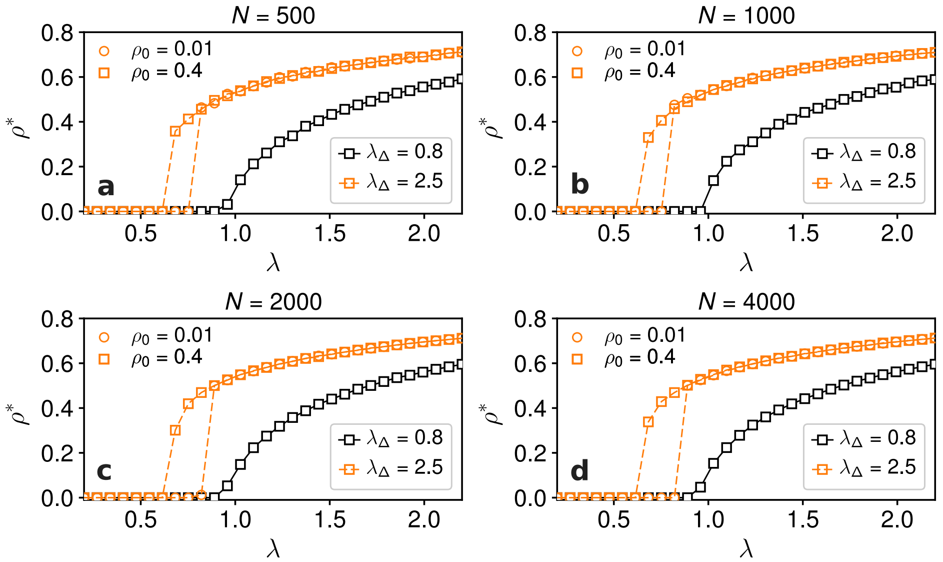


**Supplementary Figure 3.** Numerical exploration of the finite size effects on the hysteresis for a SCM of order $D = 2$ on synthetic random simplicial complexes (RSC). The RSCs are generated with the procedure described in the main text, with parameters $p_1$ and $p_\Delta$ tuned in order to produce simplicial complexes with $\langle k \rangle \sim 20$ and $\langle k_\Delta \rangle \sim 6$. Different panels correspond to different system sizes, namely (**a**) $N = 500$, (**b**) $N = 1000$, (**c**) $N = 2000$, and (**d**) $N = 4000$. Each panel shows the average stationary fraction of infected individuals plotted against the rescaled infectivity $\lambda = \beta\langle k \rangle/\mu$. The parameter $\lambda_\Delta = \beta_\Delta \langle k_\Delta \rangle/\mu$ is set to $\lambda_\Delta = 2.5$, which corresponds to the case in which we observe a discontinuous transition, with the formation of a a bistable region where healthy and endemic states co-exist and a hysteresis appears. The two types of orange symbols correspond to two different values of the initial density of infected individuals for $\lambda_\Delta = 2.5$, namely $\rho_0 = 0.01$ (circles) and $\rho_0 = 0.4$ (squares). The case $\lambda_\Delta = 0.8$, in which we observe a continuous transition with no hysteresis, is shown for reference (black squares).

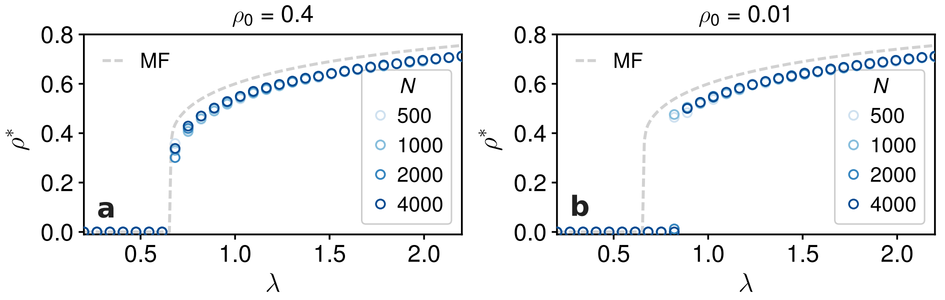


**Supplementary Figure 4.** Numerical exploration of the finite size effects on the hysteresis for a SCM of order $D = 2$ on synthetic random simplicial complexes (RSC). The two panels refer to two different values of the initial density of infected individuals, namely (**a**) $\rho_0 = 0.4$ and (**b**) $\rho_0 = 0.01$. The dashed line corresponds to the mean-field result.

## Supplementary Note 3. CASES OF HIGHER DIMENSIONS

### Case $D = 3$

Let us consider here a system with maximum dimension of simplices $D = 3$. In this case the model has three spreading parameters $\beta_1$, $\beta_2 = \beta_\Delta$ and $\beta_3$, and the evolution equation for $\rho(t)$ reads

$$d_t\rho(t) = -\mu\rho(t) + \beta\langle k\rangle\rho(t)(1-\rho(t)) + \beta_2\langle k_2\rangle\rho(t)^2(1-\rho(t)) + \beta_3\langle k_3\rangle\rho(t)^3(1-\rho(t)). \qquad (1)$$

Finding the roots of $d_t\rho(t) = 0$ yields a polynomial of degree 3, so it is possible to write these roots, corresponding to stable and unstable fixed points of the dynamics, as functions of the parameters of the model. The process is however lengthy and cumbersome, and depends moreover on three parameters, so that the representation of the whole phase diagram is not convenient.

As we want here simply to show that the phenomenology of the appearance of first order transitions obtained in the case $D = 2$, is also observed in higher dimensions, we restrict ourselves for simplicity to the case $\beta_\Delta = 0$, in which we will see that we can avoid writing the explicit solutions and resort instead to a graphical solution. This case corresponds to the hypothesis that contagion can occur only either through simple contagion or through cliques of size 4 in which 3 of the nodes are already infectious, and the evolution equation reduces to:

$$d_t\rho(t) = -\mu\rho(t) + \beta\langle k\rangle\rho(t)(1-\rho(t)) + \beta_3\langle k_3\rangle\rho(t)^3(1-\rho(t)). \qquad (2)$$

Setting $\lambda = \beta\langle k\rangle/\mu$, $\lambda_3 = \beta_3\langle k_3\rangle/\mu$ and rescaling time by $\mu$ we obtain:

$$d_t\rho(t) = \rho(t)(1-\rho(t))\left(\lambda + \lambda_3\rho^2 - \frac{1}{1-\rho(t)}\right) \qquad (3)$$

where we can define the functions $f_1(\rho) = \lambda + \lambda_3\rho^2$ and $f_2(\rho) = 1/(1-\rho)$. The sign of the temporal evolution of the density of infectious is thus given by the sign of the difference between $f_1 - f_2$. Note that $\rho(t)$ is by definition between 0 and 1 so we need to consider $f_1$ and $f_2$ only between these limits. In this interval, $f_1$ is positive and increases monotonically from $\lambda$ for $\rho = 0$ to $\lambda + \lambda_3$ for $\rho = 1$. Function $f_2$ is also positive and strictly increasing, with $f_2(0) = 1$ and $f_2$ diverging towards $+\infty$ as $\rho \to 1^-$. We also note that the equation $f_1(\rho) = f_2(\rho)$ yields a polynomial of degree 3, so it has at most 3 real roots.

Let us first consider the case $\lambda > 1$. Then at $\rho = 0$ we have $f_1 > f_2$, and as $\rho \to 1$, $f_1$ becomes smaller than $f_2$. Therefore, at small $\rho$, $d_t\rho$ is positive and hence the state $\rho = 0$ is unstable. More in detail, there are two possibilities:

- either there is one single crossing point of $f_1$ and $f_2$, at $\rho^*$. Then, $d_t\rho(t) > 0$ if $\rho(t) < \rho^*$ and $d_t\rho(t) < 0$ if $\rho(t) > \rho^*$: for any $\rho(t = 0) > 0$, the system goes to the stationary state $\rho(t \to \infty) = \rho^*$. This is similar to the usual SIS case with $\lambda_3 = 0$: the effect of a non-zero value of $\lambda_3$ is simply to shift the value of $\rho^*$.

- or there are three crossing points $\rho_1 < \rho_2 < \rho_3$. This occurs for certain combinations of values of $\lambda$ and $\lambda_3$. Then for $\rho(t) < \rho_1$, $d_t\rho(t) > 0$ so the absorbing state $\rho = 0$ is again unstable. The state $\rho_2$ is also seen to be unstable while there are two stable fixed points $\rho_1$ and $\rho_3$: depending on the value of $\rho(t = 0)$, the system will converge to one of these values.

Hence, for $\lambda > 1$, the system always reaches a stationary state with a finite fraction of infectious nodes, which in some regions of the ($\lambda$,$\lambda_3$) phase diagram, can depend on $\rho(t = 0)$.

Let us now consider the more interesting case $\lambda < 1$. Then $f_1(\rho) < f_2(\rho)$ both for $\rho = 0$ and as $\rho \to 1$. Hence $f_1 - f_2$ is negative both in 0 and 1, and either 0 or 2 of the roots of the equation $f_1(\rho) = f_2(\rho)$ are between 0 and 1. Hence, for $\rho \in [0, 1]$, either $f_1$ is always below $f_2$, or the two functions intersect in 2 points that we call $\rho_-$ and $\rho_+$ ($\rho_- < \rho_+$):

- in the former case ($f_1(\rho) < f_2(\rho)$ $\forall\rho \in [0, 1]$), $d_t\rho(t)$ is always negative so the only stationary state is the absorbing one $\rho = 0$;

- in the latter case, $d_t\rho$ is positive for $\rho(t)$ between $\rho_-$ and $\rho_+$ and negative else, so that

  - if $\rho(t = 0) < \rho_-$, $d_t\rho$ is negative, hence $\rho(t)$ decreases and the system converges to $\rho = 0$
  - if $\rho(t = 0) > \rho_-$, the system converges towards $\rho(t \to \infty) = \rho_+ > 0$.

At fixed $\lambda < 1$, the former case is obtained at small values of $\lambda_3$, while the latter is obtained for $\lambda_3$ large enough. The situation is illustrated in Fig. 5 for $\lambda = 0.5$. At the transition $\lambda_3 = \lambda_3^c$ between these two cases, $\rho_- = \rho_+ > 0$ (the functions $f_1$ and $f_2$ are tangent in this point): the transition from $\rho(t \to \infty) = 0$ for $\lambda_3 < \lambda_3^c$ to $\rho(t \to \infty) = \rho_+$ (if $\rho(t = 0) > \rho_-$) for $\lambda_3 > \lambda_3^c$ is thus a discontinuous one, in a similar way to the case $D = 2$ discussed in the main text.

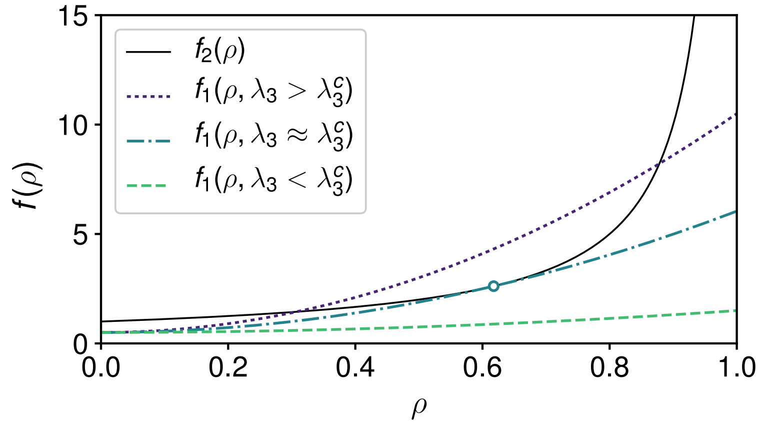


**Supplementary Figure 5.** SCM of order $D = 3$, case $\lambda = 0.5$, $\lambda_2 = 0$: $f_1(\rho)$ for various $\lambda_3$ ($<$, $\approx$ and $>$ $\lambda_3^c$), and $f_2(\rho)$. $f_1$ is below $f_2$ both at $\rho = 0$ and as $\rho \to 1$. The two curves therefore either do not cross (for $\lambda_3 < \lambda_3^c$), are tangent in $\rho_+ = \rho_-$ (for $\lambda_3 = \lambda_3^c$) or cross in two points $\rho_-$ and $\rho_+$ (for $\lambda_3 > \lambda_3^c$).

**General $D$, with $\beta_1 = \cdots = \beta_{D-1} = 0$**

For general $D$, there is no analytical solution for the stationary values of the density of infectious nodes. We show here however that, if we consider that contagion can occur only through cliques of size $D+1$, i.e., if all spreading rates $\beta_1$, $\beta_2$, ..., $\beta_{D-1}$ are null, there exists a discontinuous transition between the phase in which the spreading vanishes at low $\beta_D$ and the phase in which $\rho(t \to \infty)$ is finite at large $\beta_D$.

The evolution equation for $\rho$ reads

$$d_t\rho(t) = -\mu\rho(t) + \beta_D\langle k_D\rangle\rho(t)^D(1-\rho(t)). \tag{4}$$

Defining $\lambda_D = \beta_D\langle k_D\rangle/\mu$ and rescaling time by $\mu$ we obtain

$$d_t\rho(t) = -\ \rho(t)\Big[1 - \lambda_D\rho^{D-1}(t)(1-\rho(t))\Big]. \tag{5}$$

Defining $F_D(\rho) = 1 - \lambda_D\rho^{D-1}(1-\rho)$, we see that the sign of $d_t\rho(t)$ is opposite to the sign of $F_D(\rho(t))$, so that we need to study the sign of the function $F_D(\rho)$ for $\rho \in [0,1]$ (as the density $\rho(t)$ is by definition between 0 and 1).

We have $F_D(0) = F_D(1) = 1$. Moreover, the derivative of $F_D$ is

$$F_D'(\rho) = \lambda_D(D\rho^{D-1} - (D-1)\rho^{D-2}) = D\lambda_D\rho^{D-2}(\rho - (1-1/D)).$$

It is thus negative for $\rho < 1 - 1/D$ and positive for $\rho > 1 - 1/D$: $F_D$ first decreases as $\rho$ increases, reaches a minimum at $\rho = 1 - 1/D$ and then increases back to 1 as $\rho$ increases to 1. We have thus two cases:

- if the minimum, $F_D(1-1/D)$, is positive, then $F_D(\rho) > 0$ for $\rho \in [0,1]$: therefore, $d_t\rho(t)$ is always negative for any $\rho(t) > 0$: the density of infectious nodes can only decrease and the contagion-free state $\rho = 0$ is the only stable state.

- if instead $F_D(1-1/D) < 0$, then, as $F_D(0) = F_D(1) = 1$, by continuity the equation $F_D(\rho) = 0$ has two roots in $[0,1]$, which we call $\rho_-$ and $\rho_+$ ($\rho_- < \rho_+$). $F_D(\rho)$ is positive for $\rho \in [0,\rho_-)$ and $\rho \in (\rho_+,1]$ and negative between the two roots. Therefore
  - if $\rho(t=0) < \rho_-$, $d_t\rho(t=0)$ is negative, hence $\rho(t)$ decreases and the system converges to $\rho = 0$
  - if $\rho(t=0) > \rho_-$, the system converges towards $\rho(t \to \infty) = \rho_+ > 0$.

The condition to have $F_D(1 - 1/D) < 0$ and hence a non-trivial stationary state can be written simply as

$$1 - \lambda_D (1 - 1/D)^{D-1}(1/D) < 0$$

i.e.,

$$\lambda_D > \lambda_D^c = \frac{D^D}{(D-1)^{D-1}}.$$

Note that for $\lambda_D = \lambda_D^c$, $\rho_- = \rho_+ = 1 - 1/D$ is strictly positive, showing that the transition at $\lambda_D^c$ is discontinuous.

This shows therefore that for $\beta_1 = \cdots = \beta_{D-1} = 0$, we have the same phenomenology for any $D$ as for the case $D = 2$ studied on the main text: a discontinuous transition occurs at $\lambda_D^c = \frac{D^D}{(D-1)^{D-1}}$ between an absorbing state $\rho = 0$ and a stationary state with a non-zero density of infectious individuals $\rho_+ > 0$.

## Supplementary Note 4. HYPERGRAPHS AND SIMPLICIAL COMPLEXES

Hypergraphs are a generalization of the concept of graphs in which the edges, called hyperedges, can join any number of vertices. Formally, a hypergraph $\mathcal{H}$ is the pair of sets $(V, E)$, where $V$ is a set of vertices, and the set of hyperedges $E$ is a subset of the power set $P(V)$ of $V$. Simplicial complexes are therefore special kinds of hypergraphs, which contain all subsets of every hyperedge. A simplicial complex $\mathcal{K}$ on the set of vertices $V$ can indeed be seen as a hypergraph $\mathcal{H}$ on $V$ if the latter satisfies the extra requirement that, for each $\sigma \in E$, and for all $\nu \neq \emptyset$ such that $\nu \subseteq \sigma$, we also have $\nu \in E$. Such an extra requirement seems appropriate in the context of models of social interactions considered in our work, and it also turns useful to keep the model simple and amenable to analytical solution. However, the SCM can be straightforwardly extended to model the more general case of complex contagion processes on hypergraphs.

## Supplementary Note 5. RESULTS ON EMPIRICAL SIMPLICIAL COMPLEXES WITHOUT DATA AUGMENTATION

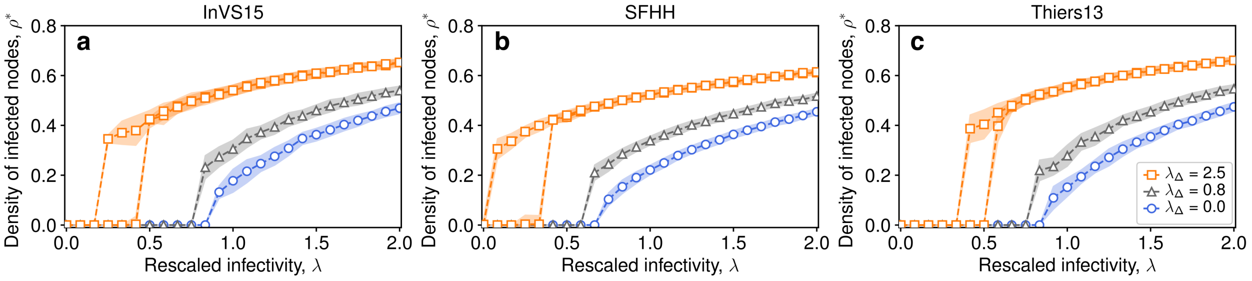


**Supplementary Figure 6.** SCM of order $D = 2$ on real-world higher-order social structures without data augmentation. Simplicial complexes are constructed from high-resolution face-to-face contact data recorded in a workplace (**a**), a conference (**b**), and a high school (**c**). The average fraction of infected nodes in the stationary state obtained numerically is plotted against the rescaled infectivity $\lambda = \beta\langle k\rangle/\mu$ for $\lambda_\Delta = 0.8$ (black triangles) and $\lambda_\Delta = 2.5$ (orange squares). The blue circles denote the simulated curve for the standard SIS model ($\lambda_\Delta = 0$), which does not consider higher order effects. For $\lambda_\Delta = 2.5$ a bi-stable region appears, where healthy and endemic states co-exist.
